# Subcellular localization of FANCD2 is associated with survival in ovarian carcinoma

**DOI:** 10.18632/oncotarget.27437

**Published:** 2020-02-25

**Authors:** Sonali Joshi, Shawn Campbell, Jeong Y. Lim, Shannon McWeeney, Adam Krieg, Yukie Bean, Nadja Pejovic, Paulette Mhawech-Fauceglia, Tanja Pejovic

**Affiliations:** ^1^Department of Obstetrics and Gynecology, Oregon Health & Science University, Portland, Oregon, 97239, USA; ^2^Division of Gynecologic Oncology, Oregon Health & Science University and Knight Cancer Institute, Portland, Oregon, 97239, USA; ^3^Biostatistics Shared Resource, Knight Cancer Institute Oregon Health & Science University, Portland, Oregon, 97239, USA; ^4^Division of Bioinformatics and Computational Biology, Knight Cancer Institute, Portland, Oregon, 97239, USA; ^5^School of Medicine, Saint Louis University, St. Louis, Missouri, 63104, USA; ^6^Sonic Healthcare USA, Austin, Texas, 78727, USA

**Keywords:** FANCD2, ovarian carcinoma, subcellular localization

## Abstract

Objective: Ovarian cancer is a leading cause of death from gynecological cancers. Late diagnosis and resistance to therapy results in mortality and effective screening is required for early diagnosis and better treatments. Expression of the Fanconi Anemia complementation group D2 protein (FANCD2) is reduced in ovarian surface epithelial cells (OSE) in patients with ovarian cancer. FANCD2 has been studied for its role in DNA repair; however multiple studies have suggested that FANCD2 has a role outside the nucleus. We sought to determine whether subcellular localization of FANCD2 correlates with patient outcome in ovarian cancer.

Methods: We examined the subcellular localization of FANCD2 in primary OSE cells from consenting patients with ovarian cancer or a normal ovary. Ovarian tissue microarray was stained with anti-FANCD2 antibody by immunohistochemistry and the correlation of FANCD2 localization with patient outcomes was assessed. FANCD2 binding partners were identified by immunoprecipitation of cytoplasmic FANCD2.

Results: Nuclear and cytoplasmic localization of FANCD2 was observed in OSEs from both normal and ovarian cancer patients. Patients with cytoplasmic localization of FANCD2 (cFANCD2) experienced significantly longer median survival time (50 months), versus patients without cytoplasmic localization of FANCD2 (38 months; *p* < 0.05). Cytoplasmic FANCD2 was found to bind proteins involved in the innate immune system, cellular response to heat stress, amyloid fiber formation and estrogen mediated signaling.

Conclusions: Our results suggest that the presence of cytoplasmic FANCD2 modulates FANCD2 activity resulting in better survival outcome in ovarian cancer patients.

## Introduction

Early stage ovarian cancer is often characterized by a lack of specific symptoms leading to a late stage diagnosis [1]. Following diagnosis, the tumor is usually first subjected to surgical cytoreduction followed by treatment with platinum based chemotherapeutic regimes [2]. Following therapy, platinum resistance is observed in ~25% of patients within 6 months [3] and at present the 5 year survival rate for ovarian cancer is 47% [4]. There is a pressing need to develop a better understanding of the molecular basis of ovarian cancer to provide the most beneficial clinical prospects, increase patient survival and decrease disease incidence. In particular, identification of specific risk factors and specific molecular defects may allow the development of patient-specific treatments for optimal survival.

Genetic and epigenetic alterations of the homologous recombination (HR) repair pathway are observed in 50% of ovarian tumors [5]. Platinum based therapy with cisplatin and carboplatin introduces intra-strand and inter-strand crosslinks (ICLs) between the purine bases of DNA, resulting in covalent tethering of both duplex DNA strands and impaired DNA replication [6]. Repair of ICL requires the intact HR pathway, which is mediated by both Fanconi anemia (FA) and *BRCA* genes [7]. Lack of HR repair sensitizes ovarian tumors to platinum-based therapeutics and therefore alteration of DNA repair genes can modulate tumor characteristics and response to therapy [8].

Fanconi Anemia (FA) is a rare autosomal recessive disease caused by germline mutations in any one of the FA complementation genes (FANC) [9]. At the cellular level FA gene deficiency causes constitutive genomic instability resulting in a predisposition to multiple cancers [10]. At present 21 human genes are associated with FA and comprise the FA complementation group proteins. Although the FA genes are phylogenetically unrelated, mutations in these genes result in a common FA phenotype implying that the proteins encoded by these genes function in a common cellular pathway [11]. DNA ICLs result in a stalled replication fork that is recognized by the FANCM–FAAP24–MHF1–MHF2 complex and is followed by the recruitment of the FA core complex [12]. The FA core complex consisting of FANC-A, -B, -C, -E, -F, -G, -L, and -M, is formed in response to DNA damage or during the S phase of the cell cycle and promotes the mono-ubiquitination and chromatin recruitment of FANCD2 and FANCI [13]. The mono-ubiquitinated FANCD2-I complex recruits DNA endonucleases and other DNA repair proteins resulting in ICL repair by homologous recombination [14]. Thus, the FA pathway plays an important role in maintaining genomic stability.

FANCD2 is a central protein in the FA pathway and aberrations in the FA genes result in defects in mono-ubiquitination of FANCD2 [15]. FANCD2 –/– mice have been reported to have an increased incidence of epithelial cancers such as breast, ovarian and liver cancers [16]. Reduced expression of FANCD2 has been reported in sporadic and hereditary breast cancer [17] and in OSE cells from women with high risk of developing ovarian cancers [18]. Interestingly overexpression of FANCD2 is also associated with worse prognosis in patients with lymph node positive colorectal cancer [19], BRCA1/2 deficient breast tumors [20], ovarian carcinoma [21] as well as metastatic melanoma [22]. Inhibition of FANCD2 expression has been correlated with resistance to multiple DNA damage inducing chemotherapeutics such as gemcitabine [23], irofulven [24]. These results suggest that FANCD2 might exhibit different functions in pre-cancer cells as compared to malignant cells. The differences in FANCD2 function may result from changes in binding partners due to post-translational modifications and/or from changes in cellular localization.

As a DNA repair protein, the function of nuclear FANCD2 has been extensively studied, but more recent studies have also shown an important role for FANCD2 in the cytoplasm. In this study, we examined the nuclear and cytoplasmic distribution of FANCD2 in ovarian cancer tissue microarray. Patients with cytoplasmic localization of FANCD2 (cFANCD2 includes patients exhibiting cytoplasmic FANCD2 staining; nFANCD2 includes patients with nuclear or no FANCD2 expression) showed increased overall survival as compared to patients with tumors expressing predominant nuclear localization of FANCD2 (nFANCD2) (*p* < 0.05). These results suggest that improved survival of patients with predominantly cFANCD2 tumor expression is either due to failure of nuclear import of FANCD2 or indeed FANCD2 has an anti-cancer role in cytoplasm.

## Results

### Nuclear and cytoplasmic localization of FANCD2

**Figure 1:**
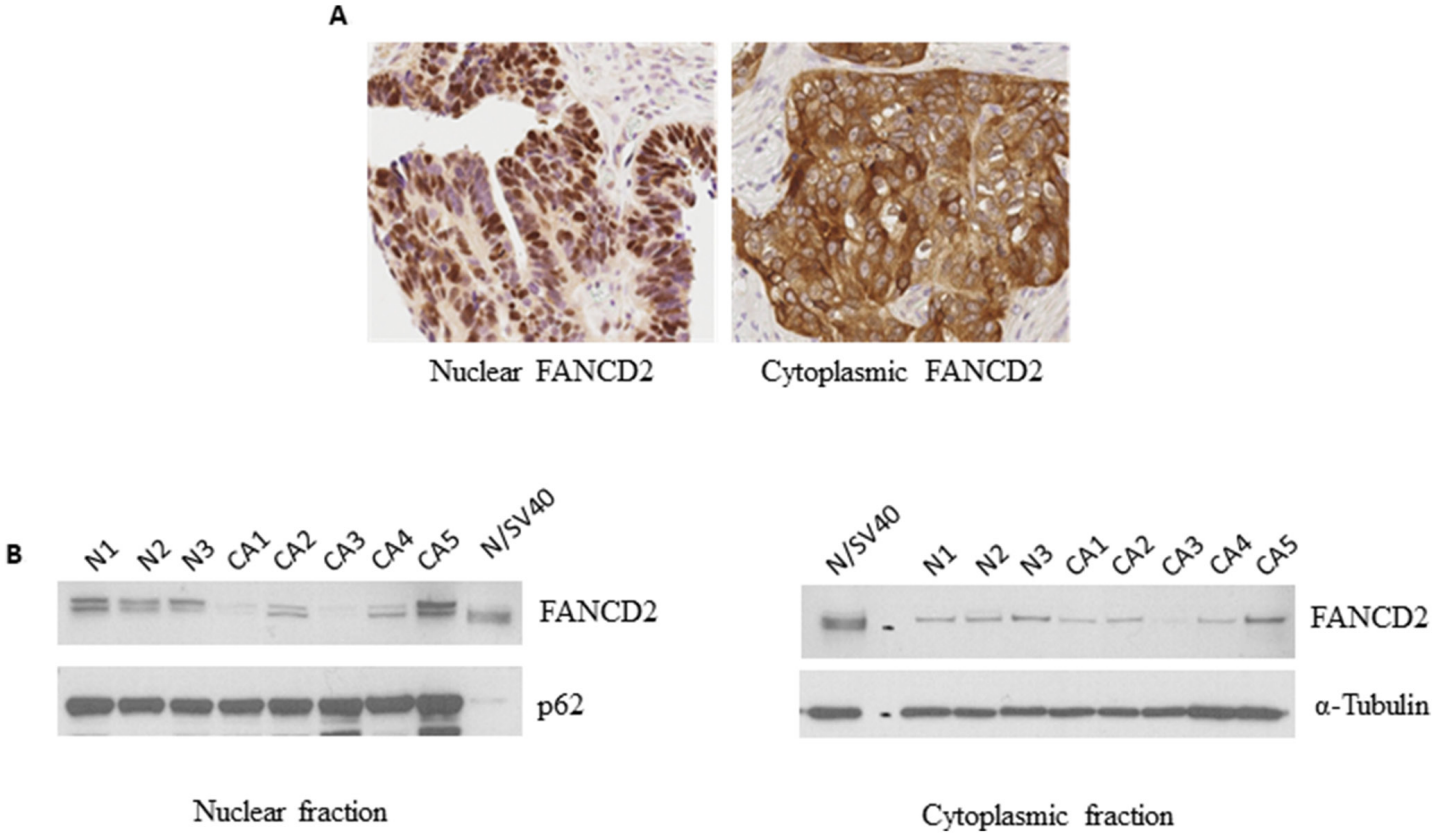
FANCD2 is present in the nucleus as well as the cytoplasm. (**A**) Representative immunohistochemistry nuclear (left) and cytoplasmic (right) staining of FANCD2 in a tissue microarray with clinically annotated ovarian tumor tissue (**B**) Nuclear (left) and cytoplasmic (right) expression of FANCD2 in primary ovarian surface epithelium cells from normal and ovarian cancer patients by Western blotting.

IHC staining of tissue microarray samples showed that FANCD2 expression is present in both the nucleus as well as the cytoplasm. A representative image of ovarian tumors with predominantly nuclear FANCD2 expression as well as predominantly cytoplasmic FANCD2 expression is shown in Figure 1A. We also examined the nuclear and cytoplasmic expression of FANCD2 *in vitro* using ovarian surface epithelial cells (OSEs) derived from normal patients (*n* = 3) or ovarian cancer patients (*n* = 5). FANCD2 expression is observed in both the nuclear and cytoplasmic fractions (Figure 1B). Mono-ubiquitination of FANCD2, which is required for its DNA repair function, was exclusively present in the nuclear fraction in OSEs from both normal patients and patients with ovarian cancer. Thus, FANCD2 expression is observed in both the nucleus and the cytoplasm.

### Cytoplasmic FANCD2 is associated with improved survival

**Figure 2:**
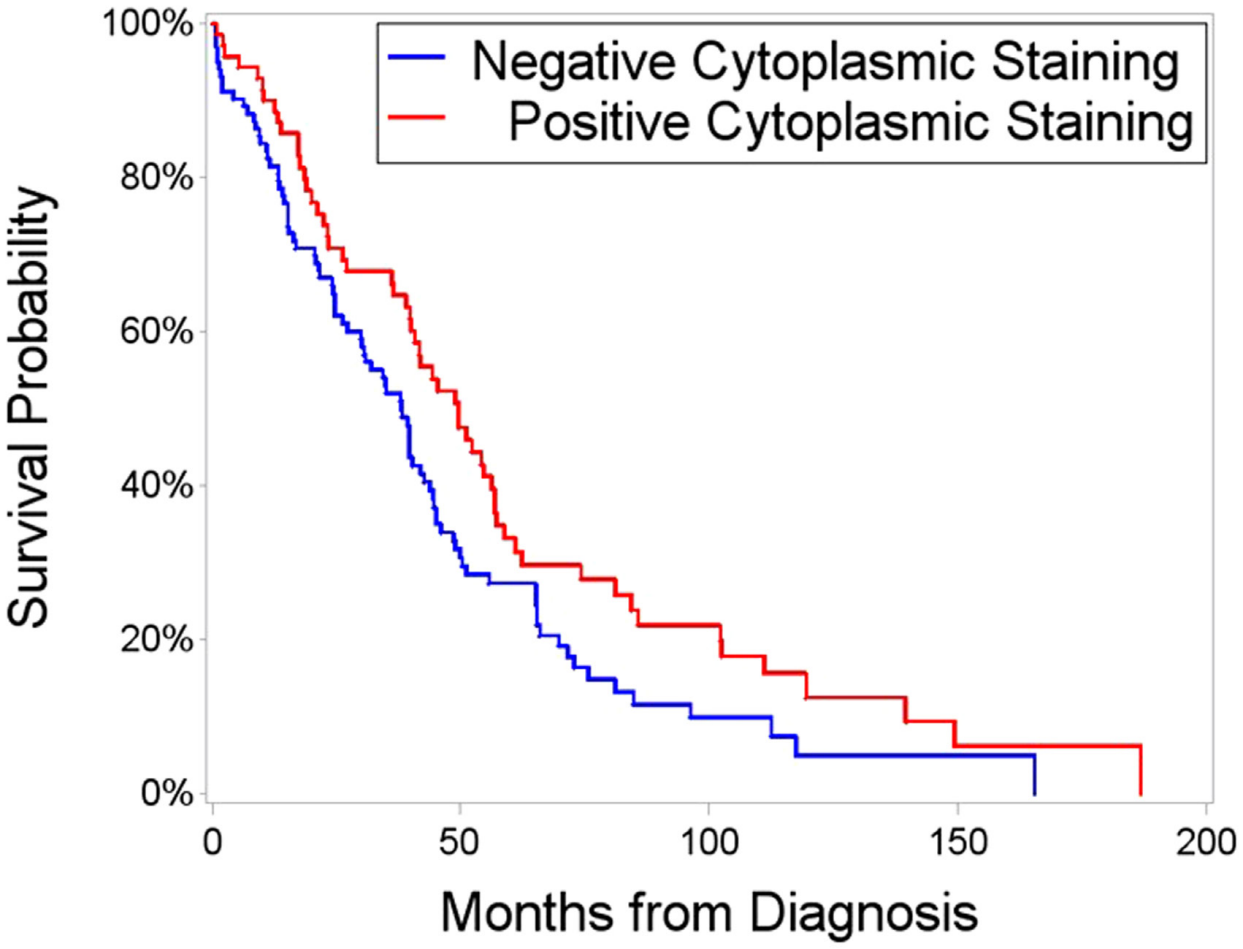
Survival time after initial diagnosis, segregated on the basis of positive versus negative cFANCD2. Red line represents patients exhibiting cytoplasmic FANCD2 staining, with a median survival of 50 months. Blue line is patients with nuclear or no FANCD2 expression; with a median survival time of 38 months. Overall survival was significantly higher (*p* = 0.0411 by Kaplan-Meier and Log-Rank test) in patients with cFANCD2.

Using a TMA with 181 patient ovarian cancer tissue samples we examined the nuclear and cytoplasmic expression of FANCD2 protein. cFANCD2 staining was observed in 73 (40.33%) of the ovarian carcinomas. There was no association between cFANCD2 staining and histotype, tumor grade, FIGO disease stage, patient age at diagnosis and cancer recurrence (Table 1). However, the presence of cFANCD2 in TMA samples was significantly associated with an increased overall survival (*p* = 0.0411). Overall median survival was 50 months in the positive cFANCD2 group and 38 months in the negative cFANCD2 group (Figure 2).

**Table 1:** Localization of FANCD2 and patient and tumor characteristics (*indicates missing data for some patients)

Characteristics	Negative FANCD2 cytoplasmic staining (*n* = 108)	Positive FANCD2 cytoplasmic staining (*n* = 73)
Histotype^*^		
**Serous Carcinoma**	84 (78.50%)	57 (78.08%)
**Endometrioid Adenocarcinoma**	7 (6.54%)	4 (5.48%)
**Clear Cell Carcinoma**	5 (4.67%)	4 (5.48%)
**Mucinous Adenocarcinoma**	2 (1.87%)	2 (2.74%)
**Undifferentiated Carcinoma**	2 (1.87%)	0 (0%)
**Poorly differentiated Carcinoma**	0 (0%)	1 (1.37%)
**Carcinosarcoma**	2 (1.87%)	1 (1.37%)
**Total**	107	73
**Fisher’s exact test**	0.95	
Tumor Grade^*^		
**Low Grade**	4 (3.81%)	7 (9.72%)
**High Grade**	101 (96.19%)	65 (90.28%)
**Total**	105	72
**Fisher’s exact test**	0.12	
FIGO disease stage^*^		
**I**	3 (2.86%)	5 (6.94%)
**II**	5 (4.76%)	5 (6.94%)
**III**	85 (80.95%)	50 (69.44%)
**IV**	12 (11.43%)	12 (16.67%)
**Total**	105	72
**Fisher’s exact test**	0.30	
**Patient’s age at diagnosis**		
**≤** **60**	55 (50.93%)	32 (43.84%)
**>60**	53 (49.07%)	41 (56.16%)
**Total**	108	73
**Chi-square test**	0.35	
Recurrence^*^		
**Yes**	91 (85.85)	62 (84.93)
**No**	15 (14.15)	11 (15.07)
**Total**	106	73
**Chi-square test**	0.86	

^*^missing information for some patients.

### Cytoplasmic binding partners of FANCD2

To identify the cytoplasmic functions of FANCD2, we immunoprecipitated cytoplasmic FANCD2 binding proteins in OSE cells from an ovarian cancer patient. The proteins identified by mass spectrometry are listed in Supplementary Table 1. The proteins bound to cFANCD2 were annotated to pathways from the Reactome Knowledgebase [25] to identify biological pathways over-represented in the proteins pulled down with cytoplasmic FANCD2. The results are presented as a Voronoi diagram (Supplementary Figure 1) providing a high-level overview. The main pathways identified were the Innate immune system, cellular response to heat stress, Amyloid Fiber formation and estrogen (ESR) mediated signaling.

A recent study by Zhang et al. (2017), identified FANCD2 binding partners by immunoprecipitation of FANCD2 from embryonic stem cells, embryos, testis and spleen derived from Flag- and hemagglutinin-tagged Fancd2 knock in mice [26]. We compared FANCD2 binding proteins identified in our analysis to FANCD2 binding partners identified by Zhang et al. to determine common proteins. The FANCD2 binding partners identified in both studies are listed in Table 2. FANCD2 was found to bind several proteins involved in the innate immune system such as HEWE1, IQGAP1, TXN, ARG1, FABP5, UBR4, KRT1 and PRSS3 in both studies.

**Table 2:** Proteins identified in our ovarian cancer patient proteomic analysis that were also reported in the Zhang et al. (2017) FANCD2 *in vivo* interaction network in one or more of four tissues: ES cells, E11.5 mouse embryos, testes and spleen mononuclear cells

Protein	ES	Embryo	Testes	Spleen
ANXA1				
HSPB1				
KRT1				
ARG1				
TXN				
PRSS3				
CAD				
IQGAP1				
FASN				
LDHA				
UBR4				
HUWE1				
FABP5				

All of these compartments express high levels of FANCD2. The Zhang protein lists were filtered to remove any proteins also seen in the control samples (same tissue from wild-type animals which do not express tagged FANCD2). Gray shading indicates shared proteins.

## Discussion

In this study, we show that cytoplasmic localization of FANCD2 is associated with positive prognosis in ovarian cancer patients. We have previously shown that FANCD2 expression is reduced in *BRCA* negative patients with a high genetic risk of developing ovarian cancer based on the patient’s personal and family history of cancer [18]. We have also shown that patients with high expression of FANCD2 have a higher risk of early recurrence of ovarian cancer [21]. In total our work suggests that FANCD2 may play a role in preventing oncogenic transformation of the OSEs but elevated expression of FANCD2 in ovarian cancer may be predictive of platinum resistance. These seemingly paradoxical observations suggest that more research is needed to delineate the role of FANCD2 in modulating ovarian cancer biology.

In breast cancer patients, lack of cFANCD2 was associated with reduced survival and co-related with a significantly higher expression of metastasis promoting proteins [27]. These observations could be a result of tumor suppressive functions of cFANCD2 or sequestering FANCD2 in the cytoplasm could suppress the activity of nuclear FANCD2. Thus, it is important to identify the extra-nuclear functions of FANCD2 and study the mechanism(s) regulating the nucleo-cytoplasmic distribution of FANCD2.

The role of FANCD2 in DNA repair has been extensively studied, but mounting evidence suggests that FANCD2 might also play a role in regulating other cellular processes. FANCD2 also plays a role in replication stress response [28]. Multiple studies have shown a role for the FA pathway in modulating cellular responses to oxidative stress; FANCD2 is also known to regulate the nuclear translocation of the phosphorylated transcription factor STAT5 in response to treatment with hydrogen peroxide, EGF and erythropoietin [29]. A study by Zhang et al. (2017) immunoprecipitated FANCD2 from multiple tissues (embryonic stem cells, embryos, testis and spleen) derived from Flag- and hemagglutinin-tagged Fancd2 knock in mice and showed that FANCD2 was associated with the mitochondrial nucleoid associated proteins Atad3 and Tufm in all tissues studied [26]. Additionally FANCD2 has also been reported to interact with ATP5α (a subunit of the mitochondrial ATP synthase) and this interaction is essential for optimal ATP production [30]. A recent study showed that genetic deletion of FANCD2 in mouse hematopoietic stem and progenitor cells enhances mitochondrial translation and exhibits increased mitochondrial respiration and increased mitochondrial reactive species [31]. Mitochondrial dysfunction has been reported to play a role in oncogenesis [32] and in response to cancer therapy [33]. Taken together these findings suggest that cytoplasmic activity of FANCD2 might play an important role in regulating its anti-tumor properties.

We also performed cFANCD2 immunoprecipitation and identified binding partners by mass spectrometry. Pathway analysis showed that FANCD2 bound several proteins involved in innate immunity. Interestingly, examining the FANCD2 binding proteins identified by Zhang et al. (2017) we identified several proteins identified by both our studies that are known to play a role in innate immunity. FA patients have been reported to exhibit attenuated immune response to viral and fungal infections [34]. A clinical report described a patient with a heterozygous mutation/deletion of FANCD2 who exhibited symptoms of combined immune deficiency in the absence of a HIV infection [35]. Future works is needed to address how FANCD2 affects immunity and our data suggests that one of the mechanisms may be mediated by cFANCD2 interaction with proteins involved in innate immunity. Our future studies will focus on validating and characterizing the interaction of proteins identified in our mass spectrometry study with FANCD2.

Nuclear localization of FANCD2 is important for its role in DNA repair. The nuclear localization signal (NLS) of FANCD2 is located in the first 58 amino acids at the N-terminal and the NLS is required for the optimal mono-ubiquitination of FANCD2 and FANCI as well as the nuclear localization of a subset of FANCI [36]. Additionally the FANCD2 NLS lies with the DNA binding domain [37]. Nuclear import of FANCD2 is mediated by its interaction with CEBPδ (CCAAT/enhancer binding protein δ) which facilitates the interaction between FANCD2 and importin 4 resulting in the nuclear localization of FANCD2 [38]. Additionally TNFα signaling has been shown to stimulate the nuclear transport of FANCD2 [39]. Mechanisms facilitating the nuclear export of FANCD2 are yet to be identified and more detailed studies are required to understand the mechanisms regulating the nucleo-cytoplasmic localization of FANCD2.

In conclusion, we have shown that cytoplasmic localization of FANCD2 suggests a favorable prognosis but the function of cFANCD2 remains to be identified. Our continued research effort is directed towards understanding the mechanisms regulating the subcellular localization of FANCD2 and the function of FANCD2 beyond DNA repair.

## Materials and Methods

### Patients and specimens

All patients underwent ovarian cancer staging and/or debulking surgery when this was considered an effective option. Tumor subtypes were classified according to 2014 World Health Organization criteria. We utilized binary grading system [21] Medical records of patients were retrospectively reviewed under an approved Institutional protocol that required written patient consent. All patients undergoing chemotherapy received platinum-based treatments as a first line treatment. Overall survival and progression times were determined, each measured from the time of diagnosis at initial surgery. Progression was defined as objective evidence of recurrence by imaging studies. The duration of overall survival was the interval between diagnosis and death. Data were censored at the last follow-up for patients with no evidence of recurrence, progression or death.

### Tissue samples and live cell collection

Tissue samples were obtained from the Oregon Ovarian Cancer Registry and Tissue Repository (OOCRTR) at the Oregon Health & Science University. Samples were paraffin-embedded and also stored in liquid nitrogen and are thus available for establishing cell cultures. All samples were collected with IRB approval.

### Tissue microarray analysis

Tissue Microarray Analysis (TMA) was performed to detect FANCD2 expression and subcellular localization in 181 paraffin-embedded samples essentially as described [21, 40]. Briefly, 0.6 mm cores were drawn from each block (donor blocks) and transferred to microarray blocks (receiver blocks). To overcome tumor heterogeneity, core samples were taken from three different areas of each tumor. Receiver blocks were sectioned, and individual sections were labeled with H&E, to identify the presence of tumor, or probed with an antibody to FANCD2 (Epitomics, San Francisco, CA), which was visualized with a peroxidase/diaminobenzidine chromatic reaction (Envision Detection System, DAKO). As a negative control, receiver block sections were also labeled without exposure to primary antibody. Subcellular localization and expression levels were determined for both cytoplasmic and nuclear fractions, by grading the staining intensity on a scale of 0–3, representing a range of background to strong signal. The grading was performed manually by an experienced Gynecologic Oncology pathologist. The extent of immunochemical reactivity was graded based on intensity as follows: 0 (background), 1 (light), 2 (moderate), 3 (strong).

### Cell culture

Cells were obtained from OOCRTR-banked, liquid nitrogen-frozen samples representing normal ovarian cells and ovarian cancer. Cultures were grown in defined medium: 50/50 DMEM/RPMI 1640, supplemented with 20% Fetal Bovine Serum (Sigma-Aldrich, St. Louis, MO) EGF (0.01 µg/ml), Gentamycin (50 µg/ml), Cipro (10 µg/ml), Insulin (10 µg/ml) and Penicillin/Streptomycin (100 µg/ml). Cells were grown to approximately 80% confluence prior to harvesting.

### Fractionation and Western blotting

Nuclear and cytoplasmic fractions were isolated using a Nuclear Extract Kit (Active Motif, Carlsbad, CA, version C4) per manufacturer’s instructions. This separates the fractions by hypotonic lysis of the cytoplasm, to isolate cytoplasmic proteins, followed by complete lysis of nuclei via detergent and denaturation of each fraction. Purified fractions were assayed for protein concentration using a Bradford-based method (BioRad) and prepared for electrophoretic separation of proteins in both nuclear and cytoplasmic fractions. Samples were denatured in reducing buffer, heated to 95°C for 10 minutes, and run on a 3–8% Tris-Acetate gel (Invitrogen; Carlsbad, CA). Following SDS/PAGE, proteins were transferred to nitrocellulose membranes, which were probed for FANCD2 (1:100) and nucleoporin 62 (1:200) (Santa Cruz Biotechnology; Santa Cruz, CA) or α-tubulin (1:900) (Sigma,-Aldrich, St. Louis, MO) as an additional loading control. HRP-conjugated secondary antibodies were then used for signal detection. Bands were visualized using Supersignal West Pico and West Femto chemiluminescence (ThermoScientific; Rockford IL) on Blue Basic Autorad film (BioExpress; Kaysville, UT).

### Co-immunoprecipitation of cytoplasmic FANCD2, mass spectrometry and proteomic analysis

Cytoplasmic fraction of FANCD2 from ovarian surface epithelial cells from an ovarian cancer patient (500 μg) was incubated with 5 μg FANCD2 antibody, Novus Biologics (Littleton, CO) in lysis buffer (50 mM Tris HCl (pH 7.5), 0.5% NP-40, 100 mM NaCl and protease inhibitor (Roche, Basel, Switzerland) overnight at 4°C. Pierce magnetic beads in H2O with 0.05% sodium azide were washed three times with wash buffer (50 mM Tris HCl pH 7.5, 0.1% NP-40, 100 mM NaCl) and incubated with FANCD2 immune complexes for 60 mins at room temperature. The beads were washed three times with wash buffer (50 mM Tris HCl pH 7.5, 0.1% NP-40, 100 mM NaCl), resuspended in sample loading buffer (106 mM Tris HCL, 141 mM Tris base, 2% LDS, 10% glycerol, 0.51 mM EDTA, 0.22 mM G250 Coomassie Blue, 0.175 mM Bromophenol Blue; pH 8.5) and were incubated at 95°C for 10 mins. The eluted proteins were separated by running on 3–8% Tris-Acetate gel (NuPage, Thermo-Fisher (Waltman, MA)) with Tris-acetate buffer (1 M Tricine, 1 M Tris base, 70 mM SDS). Bands were visualized by staining with Comassie R250 (0.1% Coomassie brilliant blue in 40% ethanol, 10% acetic acid) for one hour. Three bands (350 KDa, 260 KDa and 225 KDa) were excised, de-stained and were submitted for Liquid chromatography and tandem mass spectrometry (LC-MS/MS) to the Proteomics Shared Resource facility at the Oregon Health Science University. All peptides and corresponding spectral count results are shown in Supplementary Table 1. The peptide data was analyzed by Reactome [25].

### Statistical analysis

A total of 181 patients and tumor characteristics were summarized by FANCD2 status and compared with Fishers exact test or chi-squared test for independence as indicated. Survival analysis was conducted to determine survival difference between positive versus negative cFANCD2 using the Kaplan-Meier curve, followed by Log-rank test. Statistical significance was defined as a *p* -value of <0.05. All statistical analyses were performed using SAS statistical software (version 9.4: SAS Institute Inc, Cary, NC).

## SUPPLEMENTARY MATERIALS




