# Supplementary material for: Subcellular localization of FANCD2 is associated with survival in ovarian carcinoma
Source: Oncotarget. 2020 Feb 25;11(8):775–83. doi: 10.18632/oncotarget.27437 (PMC7055545; doi:10.18632/oncotarget.27437)
Supplement: Supplementary file 1 [file oncotarget-11-775-s001.pdf]

Subcellular localization of FANCD2 is associated with survival in ovarian carcinoma

SUPPLEMENTARY MATERIALS

Supplementary Table 1: Peptides and spectral counts of proteins identified as binding partners of cytoplasmic FANCD2 by mass spectrometry. See Supplementary Table 1

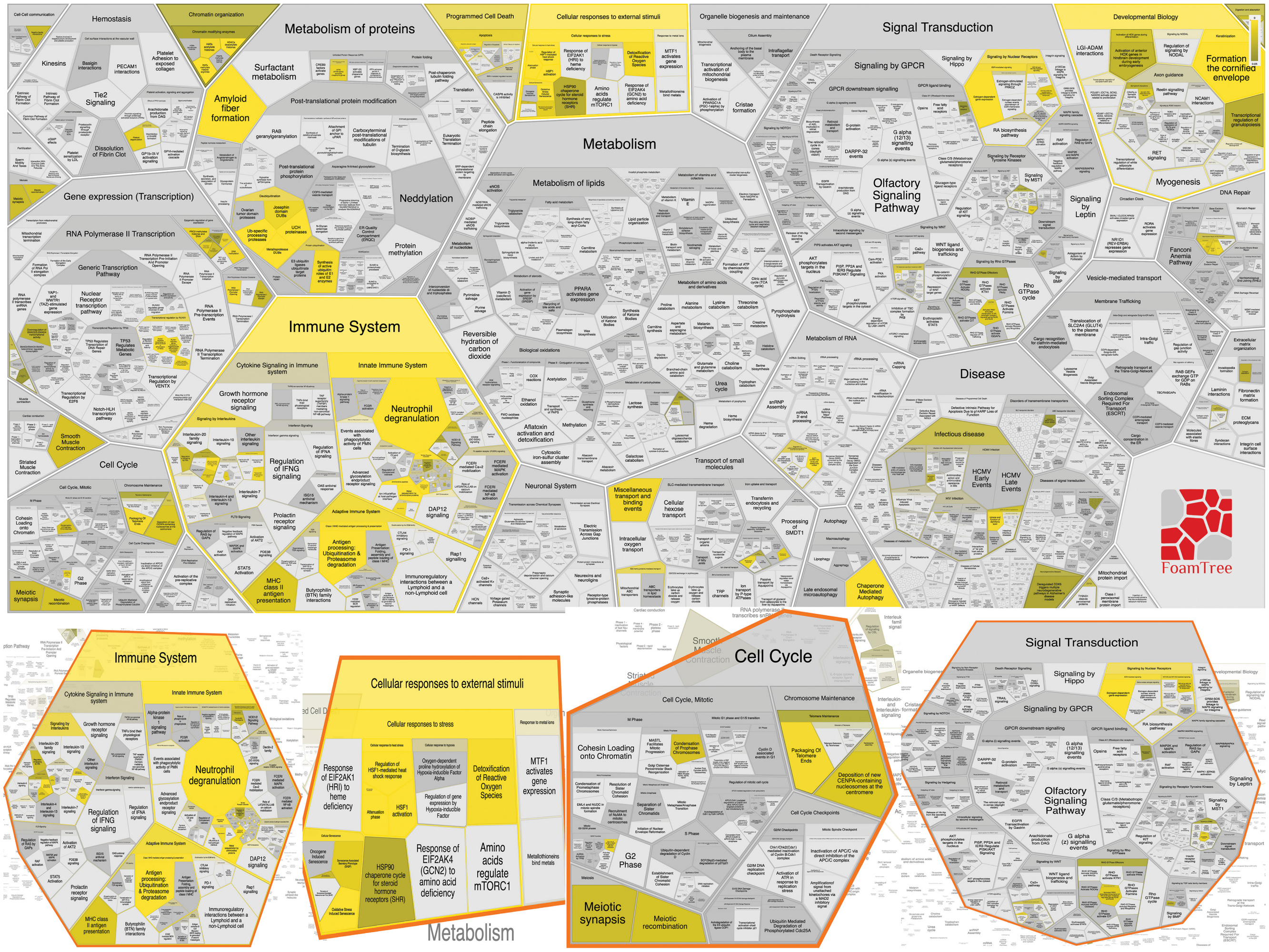

Supplementary Figure 1: A high level, genome-wide Reactome pathway overview of the pathways associated with the proteins identified in our ovarian cancer patient screen, presented as a Voronoi diagram. Enriched pathways (highlighted in yellow with adjusted *p*-value < 0.05) included Innate Immune System and child pathways like Neutrophil degranulation; cellular response to heat stress and child pathway HSF1 (a key mitotic regulator) activation and its associated pathways; Amyloid Fiber formation and ESR mediated signaling. The diagram was created using the Foamtree visualization library (https://carrotsearch.com/foamtree/).
